# β-Casein Polymorphism as a Potential Evolutionary Trade-Off: The Rise of A1 Under Intensive Selection and Its Implications for Gastrointestinal Tolerance and Agroecological Resilience

**DOI:** 10.3390/vetsci13050473

**Published:** 2026-05-13

**Authors:** András József Tóth, Szilvia Kusza, Gergő Sudár, Atilla Kunszabó, Márton Battay, Miklós Süth, András Bittsánszky

**Affiliations:** 1 Department of Food Hygiene, Institute of Food Chain Science, University of Veterinary Medicine Budapest, István u. 2., 1078 Budapest, Hungary; toth.andras.jozsef@univet.hu (A.J.T.); kunszabo.atilla@univet.hu (A.K.); battay.marton@univet.hu (M.B.); suth.miklos@univet.hu (M.S.); bittsanszky.andras@univet.hu (A.B.); 2 Centre for Agricultural Genomics and Biotechnology, Faculty of Agricultural and Food Sciences and Environmental Management, University of Debrecen, Egyetem tér 1, 4032 Debrecen, Hungary; 3 CERES Holding Ltd., 7030 Paks, Hungary; gergo.sudar@ceresholding.eu

**Keywords:** A1/A2 milk, agroecology, BCM-7, *CSN2*, dairy processing, gastrointestinal tolerance, *One Health*, traceability

## Abstract

This narrative review summarizes evidence on the bovine β-casein (*CSN2*) A1/A2 polymorphism as a case study of how intensive dairy selection and global gene flow can reshape allele frequencies in ways that matter for consumers, processing and agroecological resilience. We draw together evidence from (i) population-genetic surveys of *CSN2* in contrasting cattle populations, including a descriptive summary of published genotype-frequency studies; (ii) controlled human studies that separate A1-containing from A2-only dairy exposure; and (iii) dairy technology and the authenticity literature relevant to identity-preserved A2 value chains. Across intensively selected Holstein-Friesian populations, A1 was consistently present at substantial frequency (approximately one-third), whereas indigenous, beef and zebu-adjacent populations were typically A2-enriched, highlighting the role of historical breed formation and modern introgression in shaping apparent geographic and climatic patterns. Human intervention studies most consistently support improved short-term gastrointestinal tolerance with A2-only milk in susceptible individuals, while evidence for longer-horizon systemic outcomes remains mixed and insufficient for causal disease claims. Processing and analytical studies suggest that β-casein genotype can modestly affect coagulation and product behavior in a context-dependent manner and that validated proteoform quantification coupled with traceability is essential for credible A2 labeling at scale. We discuss implications for breeding programs, including staged A2 selection that avoids performance trade-offs, and emphasize governance of artificial insemination and supply-chain segregation as levers to limit inadvertent allele diffusion while supporting climate-relevant genetic resources in locally adapted breeds. Collectively, the reviewed evidence suggests that A1/A2 β-casein can be usefully interpreted within a *One Health* framework spanning animal genetics, dairy systems and human tolerance research.

## Simple Summary

Cow’s milk contains a major protein called β-casein, which occurs mainly in two forms, A1 and A2. This review examines how modern dairy breeding has changed the frequency of these forms in cattle populations and why this may matter for consumers, dairy production and the future resilience of livestock systems. The available evidence shows that A1 has become common in highly selected dairy breeds, especially Holstein cattle, whereas A2 remains more frequent in many traditional, indigenous and beef breeds. Studies in humans most consistently indicate that milk containing only the A2 form may cause less short-term digestive discomfort in some milk-sensitive individuals than conventional milk containing both A1 and A2. However, current evidence does not support strong conclusions about broader long-term health effects. The review also highlights that careful testing and traceability are needed to support trustworthy A2 milk production and labeling. This topic is important because it links animal breeding, food quality, consumer tolerance and the conservation of locally adapted cattle breeds that may become increasingly valuable in sustainable agriculture.

## 1. Introduction

**Figure 1:**
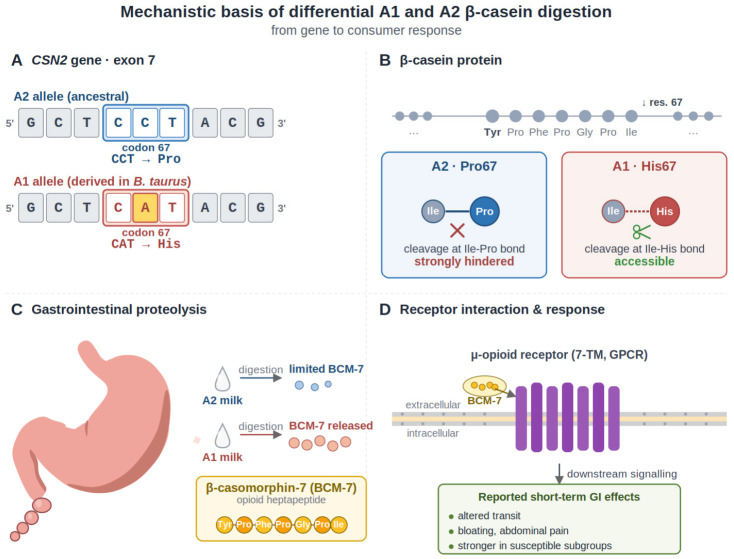
Mechanistic basis of differential A1 and A2 β-casein digestion and potential gastrointestinal response.

Polymorphism of the β-casein gene (*CSN2*) provides a useful case for examining how breeding and germplasm dissemination may reshape allele frequencies in a key nutritional trait. *CSN2* is among the most polymorphic loci affecting bovine milk proteins, and multiple protein variants have been described in cattle populations worldwide. Two variants, A1 and A2, dominate in *Bos taurus* dairy cattle and differ by a single nucleotide substitution in exon 7 of *CSN2*, replacing proline with histidine at position 67. This substitution affects proteolysis: A1 more readily releases the bioactive heptapeptide β-casomorphin-7 (BCM-7), whereas the A2 proline hampers cleavage at the relevant bond. BCM-7 generation is relevant not only to digestion but also to dairy processing, where it (or related β-casomorphins) can increase during cheese manufacture, ripening, and simulated digestion, particularly when A1 β-casein is present. BCM-7 is an opioid-like peptide capable of interacting with μ-opioid receptors, which has led to continued interest in its possible physiological effects [1,2,3] (Figure 1).

Scientific and public interest have therefore grown around the A1/A2 milk hypothesis, which proposes that A1-type β-casein consumption may be linked to human health issues, most consistently gastrointestinal effects, while broader claims remain controversial. Systematic and meta-review assessments suggest that A2-only milk may improve gastrointestinal comfort compared with milk containing A1, whereas evidence for non-gastrointestinal endpoints is inconclusive [2,4]. In a randomized, double-blind, crossover human study, only A2-type milk (versus conventional milk containing both A1 and A2) altered gastrointestinal physiology and reduced self-reported discomfort among individuals with self-reported intolerance to traditional cow’s milk [5]. Mechanistic accounts often emphasize BCM-7 and opioid signaling in the gut, but the translation from peptide release to clinically meaningful systemic outcomes is not firmly established. Consistent with this, the European Food Safety Authority (EFSA) panel concluded that available data were insufficient to establish a cause-and-effect relationship between BCM-7 (or related peptides) and the etiology/course of suggested non-communicable diseases, even as gastrointestinal effects remain central in ongoing research [2,3]. Animal-model studies continue to suggest additional mechanistic pathways; for example, prolonged exposure to diets differing in β-casein type was associated with depressive-like behavior and altered cortical μ-opioid and oxytocin receptor binding in rats [6]. Thus, whether A1 β-casein has clinically relevant adverse effects beyond gastrointestinal outcomes remains unresolved.

In this context, it is also important to understand how breeding history and selection shaped the current prevalence of A1. The A2 variant is widespread across mammals, while A1 is described as cattle-specific; modern herds commonly produce milk with mixtures of A1 and A2 due to herd composition and milk pooling [1]. Breed structure is consequential: across European dairy breeds, Guernsey is reported to have a very high A2 allele frequency (approximately 92%), whereas Holstein and Ayrshire are often reported with substantially higher A1 allele frequencies, with approximately 60% A1 reported for both Holstein and Ayrshire [1]. The 20th-century intensification of selection for yield, coupled with global dissemination of elite sires, especially Holstein Friesian, could rapidly shift allele frequencies via directional selection and hitchhiking on production-focused breeding goals [1,7]. In parallel, crossbreeding programs designed to increase yield can introduce or raise A1 frequencies in locally adapted cattle, suggesting that the rise in A1 may partly be a by-product of intensification and germplasm importation [8,9].

Relatively few studies treat β-casein variation explicitly through an evolutionary or agroecological lens, yet indigenous populations and crossbreeding dynamics are especially informative for trade-offs across environments. In Turkey, a synthesis of breed-level evidence emphasized higher A2A2 genotype frequencies in indigenous breeds and argued that local gene resources can support A2 milk production and resilience to disease pressure and adverse climate conditions, albeit typically with lower milk-yield potential than specialized dairy breeds [7]. In Indian zebu breeds, targeted genotyping likewise shows strong predominance of A2 while also challenging the assumption that zebu are uniformly A2-only: low-frequency A1 alleles were detected in Bargur and Umblachery cattle (A1 allele frequencies of 0.063 and 0.02, respectively), with no A1A1 homozygotes observed in the sampled animals [10]. These results reinforce concerns that even conservation or low-intensity systems can accumulate A1 through historical or ongoing admixture, underscoring the need to measure rather than assume β-casein status.

Beyond health and population genetics, the A1/A2 substitution can intersect with processing traits: milk-protein polymorphisms and interactions, including *CSN2* and κ-casein (*CSN3*), affect coagulation-related properties, heat stability, and yields, implying that selection for processing performance could correlate with milk-protein haplotype structure [11]. However, these technological effects should be interpreted cautiously, as the literature indicates that A2 milk may be less favorable for rennet coagulation, acid gelation, heat stability, and related processing characteristics. In controlled cheese production, semi-hard cheeses from A1A1 and A2A2 milk have been reported as broadly comparable in physicochemical quality, but with higher BCM-7 concentrations in A1A1 cheeses after pressing and during ripening [3]. Together, such findings motivate an evolutionary trade-off framing in which A1 proliferation in high-yield systems may reflect indirect selection and demographic expansion of elite lines, while A2-rich indigenous populations may reflect lineage structure and/or correlated selection for robustness under local stressors.

This review synthesizes current knowledge on the evolutionary and agroecological consequences of intensive selection for A1 β-casein. We examine how breeding practices (including crossbreeding and artificial insemination (AI)-driven dissemination) can shift A1/A2 allele frequencies and consider implications for breed diversity, resilience, and climatic adaptation. We also integrate evidence on BCM-7 formation in digestion and processing and critically summarize human and animal data on gastrointestinal and broader health-related outcomes. In addition, we address genomics and authentication issues shaping A2 value chains: genotype-based herd conversion is widely described as feasible, while analytical verification in animals and dairy matrices includes PCR-based testing, electrophoretic and chromatographic methods, spectroscopy, and immunoassays.

Beyond its relevance to dairy breeding and milk composition, the A1/A2 β-casein question can also be interpreted within a *One Health* framework. *CSN2* variation connects cattle genetics and breeding with milk supply chains, consumer exposure and the stewardship of locally adapted bovine genetic resources. This broader framing helps position A1/A2 divergence as a cross-sector issue rather than a single-trait question in dairy science alone.

## 2. Scope of the Review

This narrative review adopts a *One Health* perspective and brings together three connected strands of the literature relevant to the *CSN2* A1/A2 polymorphism: cattle population-genetic surveys, controlled human intervention studies, and dairy technology/authentication research. The relevant literature was identified from biomedical and agricultural databases, with emphasis on studies that could clarify how A1/A2 frequencies have been shaped by intensive selection and gene flow and how these patterns intersect with gastrointestinal tolerance, dairy processing, and agroecological resilience. For the cattle genetics component, particular attention was given to breed-resolved surveys reporting explicit population information and genotype or allele-frequency data. For the human evidence base, we prioritized controlled studies that clearly distinguished A2-only from A1-containing dairy exposure and reported gastrointestinal outcomes under reasonably comparable exposure conditions. Studies on processing, BCM-7 formation and authentication were synthesized qualitatively, with emphasis on experimentally transparent and practically relevant findings for identity-preserved A2 value chains.

## 3. Population Genetics of CSN2 Under Intensive Selection

### 3.1. A1 Beta-Casein in Intensively Selected Dairy Breeds

This section is based on representative genotype surveys from different cattle populations, rather than providing a global prevalence estimate. A clear production-type pattern emerged: intensively selected dairy-exotic populations generally carried higher A1 frequencies than indigenous or beef populations. Across the included strata, dairy-exotic lines typically showed A1 values around one-third (approximately 0.36), whereas indigenous taurine populations were closer to approximately 0.18 and indigenous zebu populations were typically low (approximately 0.07). These values are presented as descriptive summaries, given differences in sampling frames and reporting across surveys.

Within high-output dairy systems, Holstein Friesian repeatedly clustered at approximately one-third A1. In Ecuador, Holstein-Friesian cattle (*n* = 701) showed allele frequencies A1 = 0.372; A2 = 0.628 [12]. In Poland, a large Holstein-Friesian population (*n* = 1239) genotyped on a bovine SNP array showed A1 = 0.37; A2 = 0.63 [13] (Table 1).

Comparable A1 allele frequencies were also reported in Holstein or Holstein-Friesian populations from Italy, Australia, and the USA, with A1 values ranging from 0.304 to 0.325 [15,16,18]. New Zealand data are included as genotype-level evidence, with an A2A2 genotype proportion of 0.516 in the cited dairy cattle population [17]. These concordant values, obtained with different genotyping platforms and in different production environments, support the inference that modern Holstein lineages, expanded globally under intensive selection for milk yield, carry a stable, non-trivial A1 load.

The allele frequency does not necessarily translate linearly to the A1/A2 proteoform balance in market milk, which is directly relevant to exposure questions. In the Polish Holstein-Friesian supply zone, although genotype-derived allele frequencies suggested that A2 should dominate bulk milk (A2 = 0.63), ELISA quantification in commercial pasteurized milk and milk powder showed higher A1 than A2 variant concentrations, indicating an over-representation of the A1 proteoform in final dairy products [13]. The authors interpret this as consistent with earlier observations of unbalanced (allele-specific) *CSN2* expression and emphasize that A1 may be synthesized more efficiently, complicating attempts to reduce A1 content by selection alone [13]. This is an important mechanistic nuance for the evolutionary trade-off framing: intensive breeding can elevate A1 allele frequency, and downstream biology may further amplify A1 at the protein level in commercial products.

### 3.2. Persistence of A2 Alleles in Beef and Traditional Breeds

In contrast to dairy-exotic strata, beef and indigenous populations retained high A2 prevalence across continents. A direct example comes from China, where Angus (*n* = 85) exhibited A2 = 0.835 (A1 = 0.165) and Simmental (*n* = 201) A2 = 0.642 (A1 = 0.358) [14]. Even where Simmental is used in dual-purpose contexts, its A2 frequency exceeded many Holstein-derived intensive dairies, consistent with weaker historical selection pressure for extreme milk volume.

Indigenous and local breeds were similarly A2-rich. In Turkey, four native breeds genotyped by ACRS-PCR, namely Anatolian Black (*n* = 100), Eastern Anatolian Red (*n* = 100), Southern Anatolian Red (*n* = 87), and Turkish Gray (*n* = 87), showed tightly clustered allele frequencies around A2 ≈ 0.799–0.810 (A1 ≈ 0.190–0.201) [7]. In India, allele-specific PCR surveys found near-fixation for A2 in locally adapted cattle: Bargur (*n* = 48) had A2A2 = 87.5% and Umblachery (*n* = 42) A2A2 = 95.2% [10]. A highland-adapted population from Ladakh (*n* = 82) also remained predominantly A2 (A2 = 0.90) [9].

However, where intensive dairy germplasm is introduced, A1 can rise quickly. A small Indian market-linked sample interpreted as crossbred/admixed showed A1 = 0.365; A2 = 0.635, with no A1A1 detected but a high heterozygote proportion [8]. This pattern is consistent with A1 entering predominantly A2 backgrounds via Holstein-oriented upgrading and then persisting under continued dairy-focused selection or mating structure.

These population patterns have implications beyond descriptive genetics. Within a *One Health* framework, they help explain how breeding trajectories in cattle may influence both downstream food-system characteristics and the long-term maintenance of locally adapted animal genetic resources.

## 4. Gene Flow, Agroecology and Climate Context

Mapping breed origin/production context to broad climate strata (tropical/arid, continental, cold/highland and cosmopolitan/tropical–highland settings) revealed that apparent climate associations were strongly confounded by breed history and importation, but nonetheless informative for agroecological interpretation. In the extracted dataset, tropical/arid indigenous zebu strata (Bargur, Umblachery and other zebu breeds) were consistently A2-rich, and cold/highland Ladakhi cattle also showed high A2 frequency. In contrast, tropical–highland systems dominated by imported dairy breeds (Ecuadorian Holstein, Brown Swiss, Jersey and dairy crossbreds) tended to show more intermediate A2 levels, reflecting breed composition and recent gene flow.

A within-country geographic contrast in Ecuador illustrates how breed composition and production ecology interact: Holstein-Friesian cattle showed higher A1 allele frequency on the coast (A1 = 0.361) than in the highlands (A1 = 0.216), consistent with regional differences in herd structure and introgression patterns [12]. In parallel, continental indigenous Turkish breeds maintained an A2 allele frequency of approximately 80% despite being managed in a continental zone [7].

This combination supports the interpretation that observed climate signals largely track domestication lineages and modern gene flow (Holsteinization) rather than direct selection by temperature or aridity per se. That said, the persistence of high A2 in heat- and drought-adapted indigenous cattle (Bargur/Umblachery, and other zebu breeds) remains consistent with hypotheses that local adaptation programs seeking climate resilience can simultaneously preserve or reintroduce A2 provided that crossbreeding plans manage A1 introgression explicitly.

### Agroecological Implications and Potential Adaptive Value of A2

The pronounced breed- and region-structure in *CSN2* A1/A2 frequencies suggests that β-casein polymorphism is not merely a milk-quality issue but also a useful marker of agroecological history and gene flow. We found that A2 predominance is consistently observed in cattle populations that have been maintained under tropical, arid or otherwise stressful production environments, including indigenous *Bos indicus* and locally adapted *Bos taurus* breeds. Turkish indigenous breeds sampled across Anatolian production conditions (Anatolian Black, Eastern Anatolian Red, Southern Anatolian Red, Turkish Gray) showed broadly similar A2-dominant profiles with A2 allele frequencies of 0.799–0.810 [7]. Likewise, high-altitude Ladakhi cattle, which were kept under extreme cold, hypoxia and forage limitation, retained high A2 allele and A2A2 genotype frequencies (A2 = 0.90; A2A2 = 0.79), with the authors explicitly noting recent crossbreeding with Jersey as a feasible route for any A1 introgression into this otherwise A2-dominant population [9]. In Indian zebu breeds from Tamil Nadu, Bargur and Umblachery were near-fixed for the A2 allele, with A2 allele frequencies of 0.938 and 0.976, and A2A2 genotype frequencies of 0.875 and 0.952, respectively. The study emphasized the practical importance of screening artificial insemination (AI) sires to prevent low-frequency A1 alleles from spreading in indigenous germplasm [10]. Even in mixed-breed contexts where intensive dairy genetics have been introduced, the direction of gene flow is visible at *CSN2*: in Ecuador, imported *Bos taurus* dairy breeds and crossbreds showed moderate A1 allele frequencies, while the warmer coastal region presented higher A2 allele frequency than the cooler highlands, with A2 allele frequencies of 0.784 versus 0.639, respectively, consistent with greater zebu influence or differential breed composition across environments [12].

These associations may invite an adaptive interpretation. A key point is that the climate may be acting largely through breed history: *Bos indicus* lineages and many locally adapted cattle were developed and maintained in hot environments, while high-intensity *Bos taurus* dairy lineages expanded from continental regions. The physiological basis for this broader breed-level pattern is well established: zebu cattle exhibit superior thermotolerance at physiological and cellular levels compared with European *Bos taurus,* and warm-climate breeds show additional adaptive traits relevant to tropical production [19,20,21]. Within that context, the simplest interpretation of A2 dominance in tropical/agroecological margins is demographic and historical: A1 arose and spread mainly within European-derived dairy populations and then entered tropical systems chiefly via crossbreeding, rather than being actively filtered by climate-driven selection at the *CSN2* locus itself [9,12].

However, several hypotheses are strong enough to justify targeted testing. First, the handling and stability of milk under high ambient temperatures has long been central to pastoral systems, and casein micelle stability under heat depends on physicochemical conditions (pH, ionic balance, etc.) and protein interactions rather than on a single residue alone. Reviews of milk heat stability and heat-induced micellar transformations show that micelles are generally stable to high temperatures under normal milk conditions, but coagulation and gelation emerge when conditions shift (pH reduction, mineral equilibria, protein unfolding/aggregation) [22,23,24]. At present, there is no direct experimental basis to claim that A1 milk is intrinsically less thermostable than A2 milk in real-world tropical handling; this remains a gap. What is supported is that β-casein phenotype can alter micellar and functional attributes (mineral distribution, conformational features) and that heating can affect A1/A2 milks differently in controlled settings, with several studies reporting greater heat stability for A1A2 milk than for A2A2 milk, which could matter for processing choices in different production environments [25]. Second, if any adaptive advantage exists, it may act through calf nutrition rather than through the adult cow. Here, the evidence points toward genotype-dependent functional differences in milk coagulation rather than to a direct adaptive effect: in Holstein cows, the *CSN2* A2 allelic variant has been reported to slightly worsen rennet coagulation properties [26]. Translating cheesemaking observations to the abomasal curd formation of calves is not straightforward, but the possibility is testable: if genotype-linked differences in curd structure influence gastric emptying or peptide release kinetics in neonates, then environment (heat stress, pathogen pressure, feed scarcity) could shape selection indirectly by favoring milk that supports calf survival under those constraints. Of course, this statement is not proven, but the literature currently supports genotype effects on milk functionality and digestion-related peptide release in humans, not a demonstrated fitness advantage in calves [2]. Third, the adaptive value of A2 may be better understood as a systems trait rather than a direct climatic adaptation: A2-rich indigenous and beef populations represent reservoirs of genetic diversity that intensive dairy selection did not erode. Multiple primary surveys explicitly frame A2 predominance as a conservation and breeding opportunity, recommending routine *CSN2* screening, especially of AI bulls, in order to avoid inadvertent spread of A1 into local populations and to support identity-preserved A2 supply chains [7,9,10]. A study from Ecuador illustrates the same logic in reverse: where genotyping infrastructure and selection incentives exist, the share of A2A2 sires in imported germplasm can rise rapidly, suggesting that management decisions can reshape allele frequencies over short time horizons [12]. Thus, even if A2 is not directly heat-adaptive, it may still become agroecologically valuable under climate change because climate-resilient breeding increasingly draws on indigenous and tropically adapted cattle populations in which A2 is already common [19,20,21].

Our findings indicate that the A2 allele exhibits significant enrichment in cattle populations characterized by low-input management systems, climate stress and local adaptation. The mechanistic basis of this enrichment remains to be elucidated. It may result from direct selection on *CSN2* or may alternatively be attributable to demographic processes associated with breed history. However, the conservation implications are evident. Agrobiodiversity frameworks have effectively preserved A2-enriched genetic resources that hold potential for dual application in climate-adaptation breeding programs and differentiated dairy product markets. Realizing this potential requires that crossbreeding and artificial insemination programs incorporate active *CSN2* genotype monitoring to prevent unintended allele introgression [7,9,10].

## 5. Human Perspective: Gastrointestinal Tolerance and Beyond

Within a *One Health* framework, human gastrointestinal responses are relevant not as isolated clinical endpoints, but as downstream outcomes that may influence consumer demand, breeding preferences, and the organization of dairy value chains.

The strongest human evidence comes from controlled trials directly comparing A2-only and A1-containing milk under similar lactose exposure conditions. The landmark randomized crossover studies that most clearly define the short-term gastrointestinal tolerance signal are summarized in Table 2, including early adult and pediatric trials from China and more recent adaptation work in lactose maldigesters. These studies support a consistent pattern: in symptomatic groups, A2-only milk is generally associated with better short-term gastrointestinal tolerance than conventional milk containing both A1 and A2 β-casein.

Later studies have expanded the design space rather than simply repeating the same comparison. Some have attempted to disentangle β-casein effects from lactose maldigestion more explicitly by using lactose-matched crossover designs or alternative comparator milks. Other studies have added mechanistic endpoints such as breath hydrogen, MRI-based gastric emptying measures, stool-related outcomes, or inflammatory markers. In this respect, the human literature has moved from simple symptom comparison toward a broader question of which physiological pathways most consistently track the symptom signal [31,32].

Acute crossover studies in healthy women suggest that both comparator choice and baseline milk sensitivity influence interpretation, while newer intervention studies have begun to explore microbiome-related outcomes, repeated short-term exposure, and more heterogeneous milk-sensitive consumer groups [33,34,35,36].

Symptom improvements do not always parallel changes in inflammatory or other mechanistic biomarkers, and the field still lacks a mature body of independent, harmonized trials spanning symptoms, physiology, microbiome measures, and longer-term follow-up. Accordingly, the most defensible interpretation is that β-casein composition can influence short-term tolerance in susceptible individuals, while broader causal claims remain premature [4,37].

## 6. Processing Performance, BCM-7 Exposure and Authentication

Processing performance, BCM-7 formation, and A2 verification are discussed qualitatively, focusing on experimental evidence and reliable analytical methods. A2 raw milk should be managed as an identity-preserved material. This requires CSN2 genotyping of cows and AI sires, separate milking and storage of A2A2 milk, controlled transport and processing, batch-level records, and periodic laboratory testing. Because accidental mixing with A1/A2 or A1/A1 milk can compromise A2 claims, testing of both raw milk and finished products remains important.

The literature of technology and traceability points to three main conclusions. First, β-casein genotype, rather than BCM-7 itself, can cause small but measurable changes in milk chemistry relevant to processing, including differences in protein profile, coagulation behavior, curd firmness, and product performance. Second, BCM-7 behavior in dairy matrices depends on both genotype and processing stage. Third, analytical authentication of A2 identity preservation is increasingly feasible and important for premium-market governance.

With respect to processing performance and quality, controlled dairy studies suggest that β-casein A2 may subtly modify coagulation behavior in Holstein milk, at times in a direction viewed as less favorable for cheesemaking, most commonly through slightly longer clotting times and/or reduced curd firmness, although reported effects are modest and strongly context dependent [26]. Consistent with this, direct product-focused work in cheese indicates that many physicochemical and microbiological characteristics do not differ meaningfully between cheeses manufactured from A1A1 versus A2A2 milk, yet genotype-related divergence can still emerge in BCM-7 formation across the cheesemaking process and during ripening. In a semi-hard cheese model, BCM-7 concentrations increased over processing and were significantly higher in cheeses produced from A1A1 milk at multiple stages during pressing, after brining and throughout ripening, while broader quality metrics remained largely comparable between genotypes [3]. However, recent cheddar cheese studies indicate that genotype-dependent textural and structural differences can occur, with A2A2 cheeses reported as harder or more fracturable and structurally distinct from A1-containing cheeses after ripening [38].

From the perspective of exposure plausibility, dairy-chemistry evidence further indicates that retail milk contains only trace quantities of BCM-7 prior to digestion and that processing can influence what is analytically detectable even before gastrointestinal proteolysis takes place. In a Polish dataset using ELISA, BCM-7 levels differed by product category among processed dairy items, with the highest mean concentrations reported in UHT milk [39]. In raw milk derived from genotype-defined cows, BCM-7 values were numerically greatest in A1A2 milk, followed by A1A1, with A2A2 lowest, although absolute differences in undigested milk were small [13]. Together, these observations are compatible with the expectation that the largest A1–A2 divergence in BCM-7 exposure is most likely to arise during digestion, while also highlighting that supply-chain composition, including possible A1 over-representation at the proteoform level, may be consequential when modeling plausible exposure distributions.

Parallel advances in analytical verification underpin the practical feasibility of identity preservation. ELISA- and mass-spectrometry-based workflows have matured to quantify A1 and A2 proteoforms and associated peptides in both raw milk and processed products, and evidence from the Polish market survey suggests that bulk-milk allele frequencies may underestimate A1 proteoform prevalence in retail products, implying that direct product testing remains important when operating A2-only supply chains [13,39]. Complementing wet-lab approaches, supply-chain digitization is increasingly framed as a mechanism to strengthen identity preservation, and blockchain-enabled dairy traceability systems have been proposed and piloted for milk procurement and quality assurance [40]. Although such architectures are not inherently A2-specific, their core functionalities, batch identity, tamper-evident records and auditability map closely onto segregation requirements, particularly when paired with periodic laboratory verification. Peer-reviewed evidence supports the techno-economic plausibility of maintaining segregated A2 streams. On the demand side, consumer research identifies a distinct receptive segment (particularly milk consumers reporting discomfort) and indicates a meaningful propensity to choose A2-labeled dairy products, consistent with the existence of a differentiated A2 niche [41]. On the supply side, as genomic selection infrastructures mature and the cost–benefit balance of herd-level genotyping improves, targeted selection toward A2 becomes increasingly feasible within routine breeding workflows [42,43]. In parallel, scalable authentication tools support certification and traceability in identity-preserved chains [1], and LC–MS-based methods can discriminate β-casein genetic variants and thereby help detect admixture in segregated streams [44]. These demand- and supply-side drivers help explain why A2 certification and traceability can expand despite added operational complexity, reinforcing a pathway in which genetic selection toward A2 and verification mechanisms mutually strengthen the emerging A2 niche [11].

## 7. Evolutionary Framing: Trade-Offs and Avoidable Diffusion

The footprint of intensive selection may extend beyond genotype counts to what consumers actually receive: in Polish Holstein-Friesian cattle, although the A2 allele was more frequent than A1 (63% versus 37%), bulk milk and widely consumed milk products contained more A1 than A2 β-casein protein, consistent with unbalanced/allele-specific expression favoring the A1 variant [13]. This means that even if breeding programs increase the frequency of A2, consumer exposure to A1-derived protein and possible BCM-7 precursors in pooled dairy products may decrease more slowly than expected. This is an additional and often overlooked consequence of industrial breeding and centralized milk processing [12,39].

Paradoxically, this trade-off may not have been strictly necessary, but rather is a historically contingent outcome of breeding priorities and supply-chain structure. Evidence from production systems indicates that selecting for A2 does not inherently require sacrificing performance: in organic Holstein herds, β-casein genotype showed no meaningful disadvantage for key production traits (milk, fat, and protein yields) when compared across genotypes [18]. Likewise, in tightly controlled comparisons of cows producing similar milk yields, raw-milk sampling can be matched across *CSN2* genotypes, reinforcing that high output is compatible with A2A2 cows [39]. At the same time, the evolutionary trade-off is not only about yield versus health narrative: multiple reviews and mechanistic discussions emphasize that A2 milk is often associated with improved gastrointestinal comfort outcomes relative to A1-containing milk, while broad disease causality claims remain uncertain [2]. This health-related interpretation should also be treated cautiously, because BCM-7 concentrations in undigested milk or some processed dairy matrices may be low, matrix-dependent, and not always clearly separated between A1- and A2-related β-casein categories [13,39]. Conversely, a different but economically relevant counter-pressure may exist on the processing side: several syntheses report that A2 β-casein can be associated with poorer technological properties (like slower or weaker coagulation/curd formation) than A1-containing milks, depending on breed background and interacting protein variants [2,25]. Even within the same discussion framework, recent work highlights that A2 presence may improve nutrient recovery in cheesemaking contexts, indicating that technology trade-offs are nuanced and product-specific rather than uniformly negative or positive [39]. This reframes the historical rise in A1 as an avoidable by-product of selection priorities and supply-chain structure, rather than a biologically mandated consequence of pursuing high milk output.

**Figure 2:**
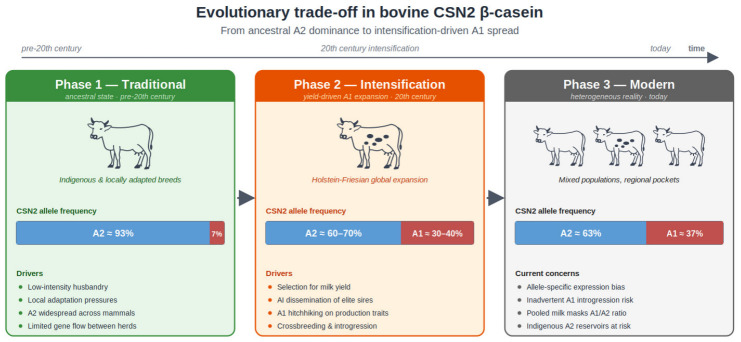
Evolutionary trade-off in bovine CSN2 β-casein. Conceptual illustration of the shift from A2-enriched locally adapted cattle populations toward increased A1 frequency under intensive dairy selection and gene flow. Allele-frequency values are approximate illustrative examples from representative studies and are not intended as universal historical or global estimates.

From an evolutionary biology perspective, the A1–A2 dynamic reinforces how artificial selection can amplify rare derived alleles far beyond what would be expected under natural or traditional low-intensity husbandry. This conceptual transition is illustrated in Figure 2, with the allele-frequency values shown as approximate examples rather than universal historical or global estimates. The A2 allele is widely described as the ancestral β-casein form, while A1 is a derived *Bos taurus* mutation (proline → histidine at position 67) that is especially prevalent in modern Holstein-Friesian cattle [13]. Comparative syntheses also emphasize that BCM-7 can be released from both β-casein variants during digestion and some dairy processes, but generally in greater amounts from A1-containing milk than from A2-only milk, supporting the idea that A1-like β-casein may contribute disproportionately to BCM-7 exposure in commercial dairy products [2].

What remains missing is a convincing fitness advantage narrative for A1 in cattle themselves: the present literature largely treats the spread of A1 as anthropogenic—driven by breed diffusion, crossbreeding, and the reproductive dominance of selected sire lines—rather than as an adaptation improving calf growth, maternal health, or resilience. Accordingly, the most parsimonious interpretation is hitchhiking under human-imposed selection regimes: we selected intensely for production and uniformity, and A1 rose incidentally because it was embedded within highly propagated genetic backgrounds, while A2 persisted where local adaptation, breed conservation, or limited introgression maintained ancestral diversity [12,45,46].

## 8. Implications for Breeding, Governance and Value Chains

### 8.1. Recommendations for Breeding Programs

In practical terms, the *One Health* value of this topic becomes most visible in breeding governance, where herd improvement, genetic resource conservation, and human-facing product expectations intersect.

Given the evidence synthesized here, a strong case emerges for incorporating *CSN2* (β-casein) genotype into routine selection and mating decisions to shift intensively managed dairy populations toward A2. Framed narrowly, this responds to a differentiated consumer market; framed more broadly, it functions as a risk-management and sustainability strategy that aligns breeding, product identity preservation, and public-facing food-quality goals [47].

Crucially, selecting toward A2 need not be paid for in lost performance. In organic Holstein systems, β-casein genotype was not a meaningful driver of production or fertility compared with herd and parity effects, and the authors noted that negative perceptions of A1 may even lead to premature culling of productive cows [18]. Population-scale selection analyses in Holsteins also show that A2/A2 frequency can rise substantially over time under intentional selection, reported as an increase from approximately 32% to 52% between 2000 and 2017, demonstrating that conversion is achievable within ongoing improvement programs rather than requiring a separate breeding reset [16]. Our own allele-frequency synthesis underscores why: even in high-output Holstein-Friesian populations, A2 remains common enough to support selection without genetic dead-ends. For example, Holstein Friesian f(A2) = 0.628 in Ecuador [12] and f(A2) = 0.63 in Polish Holstein-Friesian cattle [13], meaning a large share of animals already carry at least one A2 allele in modern intensive systems.

Operationally, the most conservative pathway for specialized dairies is directional mating without destabilizing other selection objectives: use A2A2 sires across the herd, retain replacement heifers preferentially from A2A2 dams, and phase down A1 carriers over multiple generations. Practical transition logic and do-not-disrupt herd-size considerations are explicitly described in indigenous-breed-focused selection guidance: animals with A1A1 should be excluded from replacement selection once the transition begins, while A1A2 may be retained temporarily to maintain herd size until A2A2 frequency is high enough for full conversion [7]. This kind of staged approach is particularly relevant where supply chains currently pool milk indiscriminately—because, as the Turkish analysis illustrates, even when a large A2A2 reservoir exists in local breeds, mixing can erase any A2 identity at the market level unless segregation is implemented [7]. At the same time, our results highlight why bull selection and AI governance are especially important, rather than relying on herd-level breeding alone, for limiting unintended A1 diffusion during transition [39,47].

### 8.2. Techno-Economic Innovations Enabling A2 Scaling

The expansion of A2 milk has increasingly converged with a broader digitalization of dairy genetics, quality assurance, and supply-chain governance. A recurring conclusion across the technology- and the market-focused literature is that A2 is not just a breed-genetics issue, but a segregated identity-preserved value chain that requires coordinated testing, logistics, and traceability to sustain consumer trust. Priyashantha et al. [47] explicitly frame A2 as a high-value agricultural (HVA) product whose growth depends on three infrastructural pillars: (i) validated testing, (ii) segregation from farm to processor, and (iii) credible certification and labeling systems. This matches our findings that, once A2 becomes a marketed claim, the failure mode shifts from genetics alone to mislabeling and cross-contamination risk, which must be managed through both laboratory analytics and chain-of-custody systems.

Consumer trust in A2 milk depends on verifiable authenticity and clear communication. Certification, transparent labeling, routine audits and laboratory checks can help protect the market premium. A2 milk should be presented as a differentiated option for some milk-sensitive consumers, not as evidence that conventional milk is generally harmful. On the authentication and testing side, the past few years have seen rapid methodological diversification. A2 claims are unusually challenging because A1 and A2 differ by a single amino acid, so robust detection depends on high-specificity protein/peptide assays or DNA-based methods. Recent reviews summarize a portfolio of approaches (immunoassays, FTIR/FT-NIR, and LC–MS workflows) and emphasize that uneven standardization and uneven adoption constrain international competitiveness [1,47]. Empirically, LC–MS/MS can detect trace A1 peptides even in products marketed as A2, illustrating why a genotyped herd is not sufficient proof of final-product purity [48]. In a targeted method using characteristic thermolytic peptides, small A1 β-casein signals are detected in a commercial A2 product despite manufacturer claims, highlighting real-world vulnerability to admixture or process-level mixing. Technology reviews also stress that harmonized thresholds and validated protocols are still lacking globally; market credibility depends on scalable verification methods combined with accredited testing at multiple checkpoints (farm gate, plant intake, and finished product) [47,48].

These technical requirements increasingly pull digital traceability into A2 supply chains. While blockchain is not A2-specific, food and dairy traceability reviews consistently identify it as a mechanism to improve data integrity, auditability and fraud deterrence in multi-actor supply chains, precisely the environment in which A2 identity preservation must operate [49,50]. Similarly, general food-traceability analyses describe how immutable ledgers can complement conventional quality systems by linking each batch to test results and custody events [51].

In practical terms, credible A2 markets require (i) a clear product definition, (ii) agreed testing and thresholds, and (iii) routine enforcement via accredited laboratories integrated with traceability. As more independent clinical and mechanistic evidence becomes available, the same governance infrastructure will also be important for preventing exaggerated claims. Transparent labeling should communicate only what is supported reliably by current evidence, especially findings related to gastrointestinal tolerance, and should not imply general disease prevention. This point has been emphasized in policy-oriented reviews calling for stronger evidence and standardized certification [47].

### 8.3. Reintroducing A2 Toward Sustainable Dairying

Reintroducing the A2 trait into mainstream dairy cattle can be viewed as an evolutionary restoration in the narrow sense that A2 is widely described as the ancestral β-casein form, whereas A1 is a derived *Bos taurus* mutation that reached high frequency largely through modern breed expansion [13]. From this perspective, shifting commercial herds toward A2A2 does not represent a radical re-engineering of milk, but rather a managed return toward the historical default at *CSN2* while preserving contemporary gains in productivity and efficiency. The value of this restoration is not only symbolic. It may also be relevant across three sustainability dimensions: human tolerance, genetic resources conservation, and techno-economic organization, provided that claims remain aligned with the strength of the evidence [47].

First, from the human-consumption angle, the most consistent clinical signal supporting A2 adoption concerns digestive tolerance, particularly among lactose maldigesters or self-reported milk-intolerant individuals when lactose content is held constant. In randomized, double-blind crossover designs, A2-only milk has been associated with fewer acute lactose-intolerance-like symptoms than conventional milk containing both A1 and A2 β-casein [29].

Longer short-term adaptation evidence likewise suggests symptom reductions after repeated A2 exposure versus mixed A1/A2 exposure [30]. These findings are compatible with broader mechanistic syntheses that attribute differences primarily to BCM-7-related digestion products released from A1 β-casein and to downstream effects on gut function, while also emphasizing that evidence for chronic disease endpoints remains limited and contested [2]. In sustainability terms, milk that more people can comfortably consume can be treated as a legitimate quality goal even without asserting disease prevention.

Second, A2 reintroduction can support breed diversity and climate-resilient genetic resources by attaching tangible market value to traits already common in indigenous and locally adapted populations. Reviews focused on A2 as a high-value agricultural product explicitly argue that indigenous breeds offer sustainable production pathways that can strengthen rural livelihoods while incentivizing conservation [47]. Complementing this, policy-oriented evidence from India warns that unmonitored crossbreeding and semen imports risk contamination of A2-predominant indigenous gene pools and calls for certified A2 semen and traceable supply chains to protect local genetic resources [8]. This is directly aligned with our broader agroecological interpretation: climate-adapted cattle are increasingly valuable under warming conditions, and many such populations are also A2-rich; reintroducing A2 within mainstream dairying can therefore be part of a larger strategy that values locally adapted genetics rather than replacing them.

Third, restoration has product-quality and value-chain implications that are more nuanced than the simple idea that A2 is better. On the one hand, multiple sources indicate that A2 milk may pose technological challenges in processing requiring tailored processing and careful attention to other interacting milk-protein genotypes [25,47]. On the other hand, milk-protein genotype effects are multi-locus and product-specific; recent Polish data report that nutrient recovery in cheesemaking was more efficient when the *CSN2* A2 variant was present, suggesting that processing penalties are not universal and can be managed or even offset depending on context [13]. Because A2 is a credence attribute, the sustainability case increasingly depends on credible verification: reviews emphasize certification, segregation, and traceability as prerequisites for market integrity, particularly in cooperative or smallholder scaling scenarios [8,47].

There are, of course, challenges ahead. The transition to A2 herds at scale takes time because herd turnover is biologically constrained by cow longevity and replacement dynamics. Modern dairy systems often operate with relatively short productive lifespans and substantial annual replacement rates; contemporary reviews suggest an average productive lifespan of roughly 5 years is a reasonable planning assumption in many high-producing systems, even though estimates vary by country and management [52]. Under these constraints, conversion strategies can be accelerated but not made instantaneous. Simulation and applied breeding strategy work in New Zealand indicates that combining selective use of A2A2 sires with complementary tactics can yield pure A2A2 herds within roughly 5–8 years, depending on starting structure, and sexed semen can shorten the timeline [53]. Peer-reviewed projections indicate that herd conversion toward A2A2 milk production is inherently multi-generational; for example, researchers projected that two cow generations, approximately 10 years, of mating with A2A2 bulls would increase the A2 allele frequency in cows from 0.39 to 0.85 and the A2A2 genotype frequency from 0.15 to 0.72 [54]. Farm-level analyses under bull-only strategies combined with genotyping-based replacement selection estimate a conversion period of roughly 8–15 years, depending on replacement rate and the use of sexed semen [55]. During transition, careful management is needed to avoid unintended genetic side-effects: A2-focused selection is easy genetically but can create a bottleneck if the effective pool of high-merit A2A2 sires is too narrow. Long-term selection for A2 milk can be justified if CSN2 genotype is included in a balanced breeding goal. Selection should also maintain milk yield, fertility, health, longevity and genetic diversity. If the A2 selection is too narrow, it may increase inbreeding risk or create product-specific processing constraints. Recent A2 market-and-policy syntheses explicitly caution that intense selection pressure for A2 may increase inbreeding risk unless balanced selection and diversity safeguards are built in [47].

For many Global South systems, the more urgent challenge is governance of imported genetics. Where local herds are predominantly A2, importing inexpensive A1-carrier semen can erode both product differentiation and indigenous genetic integrity. The Indian policy analysis is explicit: it highlights the lack of certified A2 semen, the risks of crossbreeding-driven A1 introgression and the need for nationwide semen-station screening and certified A2 straws as a first-line intervention [8]. In this sense, international collaboration is not only about marketing; it is about aligning breeding policy, certification and traceability so that local advantages are not unintentionally diluted.

Finally, we highlight the value of interdisciplinary approaches in dairying. The β-casein polymorphism problem spans genetics, nutrition, immunology, dairy technology, and socio-economics; correspondingly, the most integrated approaches combine balanced breeding, validated testing, and credible supply-chain governance rather than relying on any single lever. One practical research direction is the design of genuinely integrative studies. These should jointly evaluate cattle performance, milk-processing outcomes by *CSN2* genotype, and human feeding responses under standardized lactose-controlled and blinded conditions. Until then, the most defensible precautionary position is modest but actionable: moving toward A2 appears compatible with modern performance goals, offers tangible tolerance benefits for a subset of consumers, and can be structured to strengthen, rather than erode, genetic diversity and value-chain transparency, both of which are core ingredients of sustainable dairying in the 21st century [30,47].

## 9. Limitations and Research Agenda

While this paper supports the feasibility of A2-focused breeding and identity-preserved A2 dairy streams, several limitations constrain inference, especially when moving from allele-frequency patterns to evolutionary interpretation and from short-term human tolerance endpoints to long-term health claims. First, global mapping of *CSN2* A1/A2 variation remains uneven. Many genotype surveys are opportunistic (single herds/regions, limited pedigree depth), and beef, dual-purpose, and minor/indigenous breeds are still under-sampled relative to cosmopolitan dairy breeds, precisely the groups that most strongly influence our reservoir and adaptation arguments. In addition, between-study heterogeneity is amplified by inconsistent reporting (genotype counts versus allele frequencies, breed definitions, and variable quality-control detail), which limits the precision of pooled estimates and weakens climatic/ecological inferences that can be confounded by breed diffusion, historical introgression and founder effects [12]. A further technical caveat is that genotype-to-product translation is not always trivial: authentication studies emphasize that even small admixtures can matter commercially and analytically [48,56]. In addition, selective breeding focused strongly on a single allele may have unintended genetic consequences; in Australian Holstein cattle, selection for the A2 allele was associated with increased genome-wide and chromosome 6 inbreeding in A2A2 animals, alongside genotype-related differences in estimated breeding values for production, fertility, and survival traits [16].

These realities argue for coordinated, standardized surveillance of *CSN2* across production types and regions, coupled with transparent quality-control (QC) reporting, to reduce bias in any future meta-regressions linking allele frequencies to climate, management, or productivity strata.

On the human side, evidence is strongest for short-term digestive outcomes and weaker for longer-horizon disease endpoints. Controlled trials typically enroll modest samples, use relatively brief interventions (days to weeks), and rely heavily on symptom scales, with variable degrees of blinding and heterogeneity in participant selection (self-reported intolerance versus confirmed lactose maldigestion) [29,30,32]. Importantly, several trials demonstrate symptom differences without consistent changes in systemic inflammatory or oxidative-stress markers over the same time frame, underscoring that improved tolerance does not automatically imply disease modification [30]. Moreover, funding and competing-interest structures can introduce subtle risk-of-bias signals in this literature; for example, the widely cited Chinese RCT reported multiple physiological and cognitive endpoints and disclosed A2 Milk Company involvement [5]. This does not invalidate findings, but it strengthens the case for independent replication using harmonized outcomes.

Mechanistically, the microbiome and host peptidase biology are plausible effect modifiers, but direct human evidence remains sparse. Preclinical work shows that exposure to milk containing A1/A2 β-casein can shift gut microbial composition and metabolite profiles alongside neurochemical changes—supporting a gut–brain-axis hypothesis that is biologically coherent but not yet validated as a dominant pathway in humans [6]. In humans, some trials report stool-related outcomes and other gastrointestinal endpoints that may be compatible with microbiome-related differences, but microbiome sequencing and functional metagenomics are still rarely incorporated as primary outcomes [5,32]. At the same time, enzymatic clearance of opioid peptides provides a second, highly plausible layer of inter-individual variation: dipeptidyl peptidase-4 (DPP4) can degrade BCM-7, and animal work links A1 exposure to differences in DPP4 activity and inflammatory status [37,57]. However, dedicated human genetics studies (genome-wide association study (GWAS) or well-powered candidate-gene designs) that test whether DPP4 variation, opioid-receptor pathway variation or barrier-function loci predict symptom responses to A1/A2 interventions are not yet a mature evidence stream; current reviews largely position this as a priority research gap rather than an established explanatory framework [37].

Finally, it should be emphasized that removing A1 β-casein exposure is not a comprehensive strategy for preventing multifactorial diseases. Further research is needed on both processing and long-term consumer outcomes. Processing studies should test how CSN2 genotype affects heat treatment, fermentation, cheese ripening and product quality. Independent long-term human studies are also needed to define which consumers may benefit most from A2 milk. The most defensible near-term public-health and industry rationale for A2 programs is therefore narrower but tangible: improved consumer tolerance and satisfaction for a subset of milk-avoiding or milk-sensitive individuals under controlled lactose conditions, alongside a market-driven incentive to maintain rigorous traceability [1,30,32].

## 10. Conclusions

**Figure 3:**
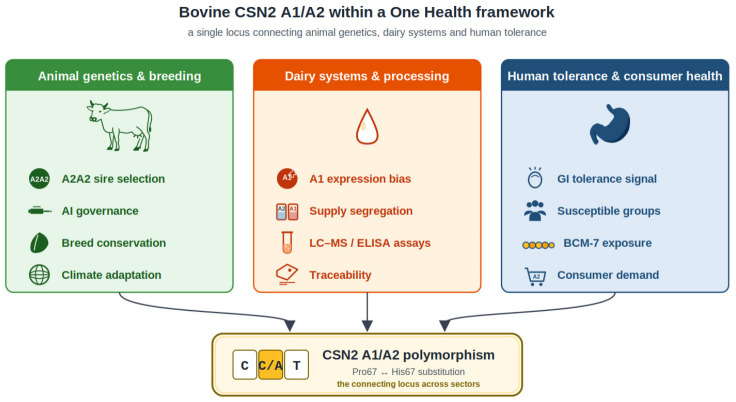
Bovine CSN2 A1/A2 variation within a One Health framework. Conceptual summary showing how CSN2 A1/A2 β-casein polymorphism links animal genetics and breeding, dairy-system governance and processing, and human tolerance and consumer-health outcomes.

Polymorphism of β-casein provides a clear example of how modern breeding objectives, artificial insemination and globalized cattle movement can shift a nutritionally salient locus rapidly and unevenly across regions. The compiled genotype surveys indicate that A1 has become common in intensively selected Holstein-Friesian systems, while many indigenous and beef-oriented populations remain A2-enriched, underscoring the importance of breed history and gene flow when interpreting geographic or climate-associated patterns. In humans, the most reproducible clinical signal favoring A2 adoption concerns short-term gastrointestinal tolerance in susceptible individuals; evidence for broader chronic disease claims remains insufficient for causal inference and should not be used for health-prevention marketing. Responsible A2 market development should combine authenticity control with balanced communication that does not undermine the established nutritional value of conventional milk. From a dairy-industry standpoint, A2 identity-preserved value chains depend on the joint implementation of genetics (staged A2 selection that avoids narrowing the breeding goal), validated analytical verification (proteoform quantification and relevant peptide assays) and supply-chain governance (segregation and traceability). Aligning A2-based breeding with conservation of locally adapted breeds may create a practical incentive to maintain climate-relevant genetic resources, provided that imported elite genetics are managed to prevent inadvertent erosion of A2-rich populations. The A1/A2 β-casein issue is best understood not only as a question of milk-protein polymorphism, but also as a *One Health* topic situated at the interface of animal breeding, human dietary response, and the stewardship of bovine genetic resources. This perspective helps explain why *CSN2* variation matters simultaneously to cattle populations, dairy value chains, and consumers. This cross-sector interpretation is summarized in Figure 3, which places CSN2 A1/A2 variation within a One Health framework linking animal genetics, dairy systems and human tolerance.

## Figures and Tables

**Table 1:** *CSN2* (A1/A2) frequencies reported in key cattle populations highlighted in this work.

Population	Country	Production Context	n	*CSN2* Allele Frequency (95% CI)	References
Holstein Friesian	Ecuador	Commercial dairy	701	A1 = 0.372 (0.347–0.397); A2 = 0.628 (0.603–0.653)	[12]
Brown Swiss	Ecuador	Commercial dairy	230	A1 = 0.357 (0.314–0.401); A2 = 0.643 (0.599–0.686)	[12]
Jersey	Ecuador	Commercial dairy	86	A1 = 0.273 (0.212–0.344); A2 = 0.727 (0.656–0.788)	[12]
Gyr	Ecuador	Commercial dairy	258	A1 = 0.145 (0.118–0.178); A2 = 0.855 (0.822–0.882)	[12]
Cross-bred	Ecuador	Commercial dairy	324	A1 = 0.356 (0.321–0.394); A2 = 0.644 (0.606–0.679)	[12]
Holstein Friesian	Poland	Commercial dairy	1239	A1 = 0.370 (0.352–0.390); A2 = 0.630 (0.610–0.648)	[13]
Simmental	China	Beef (seedstock)	201	A1 = 0.358 (0.313–0.406); A2 = 0.642 (0.594–0.687)	[14]
Angus	China	Beef (seedstock)	85	A1 = 0.165 (0.117–0.228); A2 = 0.835 (0.772–0.883)	[14]
Anatolian Black	Turkey	Native (multipurpose)	100	A1 = 0.200 (0.150–0.261); A2 = 0.800 (0.739–0.850)	[7]
Eastern Anatolian Red	Turkey	Native (multipurpose)	100	A1 = 0.195 (0.146–0.255); A2 = 0.805 (0.745–0.854)	[7]
Southern Anatolian Red	Turkey	Native (multipurpose)	87	A1 = 0.190 (0.148–0.267); A2 = 0.810 (0.733–0.852)	[7]
Turkish Gray	Turkey	Native (multipurpose)	87	A1 = 0.201 (0.150–0.261); A2 = 0.799 (0.739–0.850)	[7]
Bargur	India	Indigenous zebu	48	A1 = 0.062 (0.029–0.130); A2 = 0.938 (0.870–0.971)	[10]
Umblachery	India	Indigenous zebu	42	A1 = 0.024 (0.007–0.083); A2 = 0.976 (0.917–0.993)	[10]
Ladakhi	India	Indigenous high-altitude	85	A1 = 0.100 (0.063–0.154); A2 = 0.900 (0.846–0.937)	[9]
Crossbred (Port Blair)	India	Crossbred farm herd	26	A1 = 0.365 (0.248–0.501); A2 = 0.635 (0.499–0.752)	[8]
Holstein-Friesian cattle	Italy	Commercial dairy	1629	A1 = 0.304 (0.288–0.320); A2 = 0.607 (0.590–0.623)	[15]
Holstein cattle	Australia	Commercial dairy	69,753	A1 = 0.325 (0.322–0.327); A2 = 0.675 (0.673–0.678)	[16]
Dairy cattle	New Zealand	Commercial pasture-based dairy herds	657	A2A2 genotype proportion = 0.516 (0.478–0.554)	[17]
Holstein cattle	USA	Certified-organic dairy herds	1982	A1 = 0.322 (0.308–0.337); A2 = 0.678 (0.663–0.692)	[18]

**Table 2:** Controlled human trials comparing A1-containing versus A2-only milk.

References	Population/Design	Exposure	Lactose Control *	Main GI Finding
[5]	Adults with self-reported intolerance; DB crossover; *n* = 45	250 mL after two meals daily for 14 days	±	Conventional A1/A2 milk worsened PD3 symptoms and prolonged gastrointestinal transit, whereas A2-only milk did not aggravate PD3 symptoms relative to baseline.
[27]	Chinese adults; multicentre DB crossover; *n* = 600	Single 300 mL exposure per period	+	All six GI symptom scores were lower with A2 milk at 1 and 3 h; at 12 h, bloating, abdominal pain, stool frequency, and stool consistency remained lower than with conventional milk.
[28]	Preschool children with milk intolerance; multicentre DB crossover; *n* = 75	150 mL twice daily after a meal for 5 days	?	A2-only milk reduced GI symptom severity, lowered stool frequency, improved stool consistency, and improved Subtle Cognitive Impairment Test (SCIT) accuracy versus conventional milk.
[29]	Lactose maldigesters/intolerant adults; DB crossover; *n* = 33	Four milk challenges after an overnight fast (A2-only, Jersey, conventional, lactose-free); symptoms and breath hydrogen followed for 6 h	+	In verified lactose-intolerant subjects, A2-only milk reduced abdominal pain versus conventional milk; in the all-maldigesters analysis, A2-only milk also lowered combined symptom scores and hydrogen production.
[30]	Confirmed lactose maldigesters; DB crossover; *n* = 16	250 mL twice daily with meals for 2 weeks per period; minimum 6-day washout; same-milk challenge on day 15	+	During the 2-week intervention, A2 milk was associated with lower fecal urgency; after the intervention, bloating and flatulence during the same-milk challenge were lower with A2. Hydrogen, immunoglobulin G1 (IgG1), high-sensitivity C-reactive protein (hsCRP), and glutathione (GSH) were not significantly different, while total IgG differed in one comparison.

* Lactose control: +, explicit or closely matched lactose exposure; ±, products described as identical or composition-matched but lactose not separately quantified; ?, not verifiable from the main report. DB, double-blind.

## Data Availability

Not applicable.

## Abbreviations

The following abbreviations are used in this manuscript:

A1/A2	beta-casein A1 and A2 variants
ACRS-PCR	amplification created restriction site polymerase chain reaction
AI	artificial insemination
BCM-7	beta-casomorphin-7
*CSN2*	beta-casein gene
*CSN3*	kappa-casein gene
DB	double-blind
DNA	deoxyribonucleic acid
DPP4	dipeptidyl peptidase-4
EFSA	European Food Safety Authority
ELISA	enzyme-linked immunosorbent assay
FTIR	Fourier-transform infrared spectroscopy
FT-NIR	Fourier-transform near-infrared spectroscopy
GI	gastrointestinal
GSH	glutathione
GWAS	genome-wide association study
hsCRP	high-sensitivity C-reactive protein
HVA	high-value agricultural
IgG	immunoglobulin G
IgG1	immunoglobulin G1
LC-MS	liquid chromatography–mass spectrometry
LC-MS/MS	liquid chromatography–tandem mass spectrometry
MRI	magnetic resonance imaging
PCR	polymerase chain reaction
PD3	post-dairy digestive discomfort
QC	Quality Control
RCT	randomized controlled trial
SCIT	Subtle Cognitive Impairment Test
SNP	single nucleotide polymorphism
UHT	ultra-high temperature
